# Macrophage‐1 antigen exacerbates histone‐induced acute lung injury and promotes neutrophil extracellular trap formation

**DOI:** 10.1002/2211-5463.13779

**Published:** 2024-02-15

**Authors:** Tomohiro Mizuno, Fumihiko Nagano, Kazuo Takahashi, Shigeki Yamada, Kazuhiro Fruhashi, Shoichi Maruyama, Naotake Tsuboi

**Affiliations:** ^1^ Department of Pharmacotherapeutics and Informatics Fujita Health University School of Medicine Toyoake Japan; ^2^ Department of Nephrology Nagoya University School of Medicine Japan; ^3^ Department of Biomedical Molecular Sciences Fujita Health University School of Medicine Toyoake Japan; ^4^ Department of Nephrology Fujita Health University School of Medicine Toyoake Japan

**Keywords:** acute lung injury, extracellular histone, macrophage‐1 antigen, NETosis, platelet‐leukocyte aggregates

## Abstract

Acute lung injury (ALI), which occurs in association with sepsis, trauma, and coronavirus disease 2019 (COVID‐19), is a serious clinical condition with high mortality. Excessive platelet‐leukocyte aggregate (PLA) formation promotes neutrophil extracellular trap (NET) release and thrombosis, which are involved in various diseases, including ALI. Macrophage‐1 antigen (Mac‐1, CD11b/CD18), which is expressed on the surface of leukocytes, is known to promote NET formation. This study aimed to elucidate the role of Mac‐1 in extracellular histone‐induced ALI. Exogenous histones were administered to Mac‐1‐deficient mice and wild‐type (WT) mice with or without neutrophil or platelet depletion, and several parameters were investigated 1 h after histone injection. Depletion of neutrophils or platelets improved survival time and macroscopic and microscopic properties of lung tissues, and decreased platelet‐leukocyte formation and plasma myeloperoxidase levels. These improvements were also observed in Mac‐1^−/−^ mice. NET formation in Mac‐1^−/−^ bone marrow neutrophils (BMNs) was significantly lower than that in WT BMNs. In conclusion, our findings suggest that Mac‐1 is associated with exacerbation of histone‐induced ALI and the promotion of NET formation in the presence of activated platelets.

## Graphical abstract

Macrophage‐1 antigen (Mac‐1) deficiency ameliorated histone‐induced acute lung injury (ALI) and myeloperoxidase elevation in plasma, and decreased the formation of neutrophil extracellular trap (NET)‐like chromatin fibers in the presence of activated platelets. Thus, Mac‐1 is associated with the exacerbation of histone‐induced ALI and the promotion of NET formation in the presence of activated platelets.

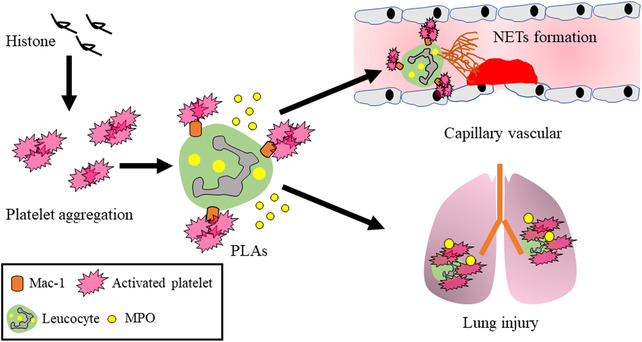

**Abbreviations**
ALI: acute lung injury
BMNs: bone marrow neutrophils
CLP: cecal ligation and puncture
COVID‐19: coronavirus disease 2019
DAMPs: damage‐associated molecular patterns
GPIbа: Glycoprotein Iba
HMGB1: high mobility group box 1
Mac‐1: macrophage‐1 antigen
MPO: myeloperoxidase
NE: neutrophil elastase
NETs: neutrophil extracellular traps
PAMPs: pathogen‐associated molecular patterns
PLAs: platelet‐leukocyte aggregates
PRP: platelet‐rich plasma
WT: wild‐type

Acute lung injury (ALI), which occurs in association with sepsis, trauma, and coronavirus disease 2019 (COVID‐19), is a serious clinical condition with high mortality [1, 2, 3]. Alveolar injury interrupts the regulation of fluid movement between the interstitium and alveoli, eventually leading to lung edema and impaired gas exchange [4]. These symptoms develop due to the highly complex coagulation cascade and complement system [2]. Excessive activation of the immune and coagulation systems increases the production of damage‐associated molecular patterns (DAMPs) and several molecules, including plasminogen activator inhibitor‐1 and IL‐1β, due to platelet activation [5, 6]. Moreover, neutrophil extracellular traps (complexes of chromosomal DNA, histones, and granule proteins, which are released by neutrophils), are increased in the plasma and bronchoalveolar lavage fluids of patients with ALI [7, 8], promoting neutrophil migration [9], platelet aggregation [10], and vascular endothelial damage [11] in lung tissues. Lung epithelial damage induced by extracellular histones leads to abnormalities of gas exchange.

Activated leukocytes interact with platelets and play a role in host immunity and hemostasis; however, excessive platelet‐leukocyte aggregate (PLA) formation promotes neutrophil extracellular trap (NET) release and thrombosis, which are involved in various diseases, including glomerulonephritis, chronic liver disease, and ALI [12]. Macrophage‐1 antigen (Mac‐1, CD11b/CD18), which is expressed on the surface of leukocytes, promotes PLA formation via receptors such as glycoprotein (GP) Ibα and GPIIb/IIIa [13, 14]. In addition, Mac‐1 has many other functions, including leukocyte recruitment [15] and the promotion of NET formation in response to infection with *Aspergillus fumigatus* [16]. The role of Mac‐1 in cecal ligation and puncture (CLP)‐induced sepsis has been previously explored. Mac‐1‐deficient mice showed high mortality due to CLP‐induced sepsis [17], whereas immunoneutralization of Mac‐1 in wild‐type (WT) mice alleviated CLP‐induced lung edema [18] and anti‐Mac‐1 designed ankyrin repeat protein (DARP) ameliorated ALI [19], suggesting that Mac‐1 promotes lung injury induced by pathogen‐associated molecular patterns (PAMPs). Nevertheless, few studies have focused on the role of Mac‐1 in lung injury induced by DAMPs.

Due to the COVID‐19 pandemic, the number of patients with ALI is increasing worldwide. Therefore, although the epithelial repair program in ALI has been elucidated [20], development of new therapeutic strategies is needed. NETs are recognized as key products driving COVID‐19‐based immunothrombosis, and NET‐containing microthrombi with neutrophil–platelet infiltration are enriched in the lung tissues of patients with COVID‐19 [8, 21]. Therefore, elucidating the mechanisms underlying DAMP‐induced lung injury would contribute to developing new therapeutics of COVID‐19. Moreover, extracellular histone is a type of DAMP released via the NETosis of dying cells. The present study aimed to reveal the role of Mac‐1 in DAMP‐induced lung injury by evaluating the mortality and pathological findings in a histone‐induced ALI mouse model.

## Materials and methods

### Animals

All animal experiments in this study were approved by the Institutional Animal Care and Use Committee of Nagoya University School of Medicine (approval number: 20353) and performed in accordance with the relevant guidelines and regulations, including the ARRIVE guidelines. Nine to twelve‐week‐old male Mac‐1 knockout (Mac‐1^−/−^) C57BL/6 mice were obtained from T. N. Mayadas, and C57BL/6 WT mice were purchased from Japan SLC (Shizuoka, Japan). All mice were maintained in virus‐ and antibody‐free facilities and given free food and water access. The histone‐induced ALI model was constructed as previously described [22]. Briefly, WT and Mac‐1−/−mice received a single tail vein injection of calf thymus histone which contains histone H3, H4, H2A, and H2B (60 μg·g^−1^; Sigma‐Aldrich, St Louis, MO, USA) and were monitored for up to 1 h for survival time analysis. The survival analysis was independently conducted before other experiments.

### Histological analysis

We conducted a histological analysis of lung injury according to previously described methods [22]. Briefly, lung tissues were collected from anesthetized mice 1 h after histone injection (60 μg·g^−1^) and weighed, after which the tissue samples were embedded in OCT compound (Sakura Fine Technical, Tokyo, Japan) and frozen in liquid nitrogen. Thereafter, lung tissues (10 μm thick) were prepared using a microtome and stained with hematoxylin and eosin. Whole section images were captured at 40× magnification using a BZ‐X800 fluorescence microscope (Keyence, Osaka, Japan). To evaluate the extent of pulmonary hemorrhaging, we measured the proportion of the bleeding area using bz‐x analysis software (Keyence).

### Platelet counts and flow cytometry

Blood samples were collected from anesthetized mice 1 h after histone injection (60 μg·g^−1^), after which they were mixed with EDTA‐2K (Dojindo Molecular Technologies, Kumamoto, Japan) and used for platelet counts. Custom platelet count measurements were conducted by Sanritsu Zelkova Co. Ltd. (Kanagawa, Japan) using whole blood samples.

Blood samples were mixed with 3.13% (w/v) sodium citrate (Sigma‐Aldrich) and used for flow cytometry. PLAs were determined as previously described [23]. Briefly, each blood sample was mixed with PE‐conjugated rat anti‐mouse CD45 antibody (clone 30‐F11; BioLegend, San Diego, CA, USA) and BV421‐conjugated rat anti‐mouse CD41 antibody (clone MwReg30; BioLegend), or isotype control. To block non‐specific binding and remove dead cells from the analysis, each mixture was added to a rat anti‐mouse CD16/CD32 monoclonal antibody (clone 2.4G2; BD Biosciences, Franklin Lakes, NJ, USA) and a Zombie NIR™ Fixable Viability kit (BioLegend). The mixture was then incubated for 15 min at room temperature (18–25 °C) in the dark. After fixation with FACS lysing solution (BD Biosciences), the number of CD45^+^CD41^+^ cells was determined using an LSR Fortessa™ X‐20 system (BD Biosciences). Finally, the percentage of PLAs was calculated using flowjo software (Tree Star Inc, San Carlos, CA, USA).

### Enzyme‐linked immunosorbent assay (ELISA) of myeloperoxidase (MPO)

We obtained plasma by centrifugation at 1500 ***g*** for 10 min at 4 °C. Plasma MPO levels were measured using a Mouse Myeloperoxidase ELISA kit (Thermo Fisher Scientific, Waltham, MA, USA) according to the manufacturer's instructions.

### Neutrophil and platelet depletion

Neutrophil and platelet depletions were conducted to clarify the function of neutrophils and platelets in our experimental ALI model. We intravenously injected anti‐Ly6G antibody (Clone 1A8; Bioxcell, NH, USA; 250 μg/body) or rat IgG2 isotype antibody (250 μg/body; Bioxcell, Lebanon, NH, USA) into WT mice 48 h before histone injection (60 μg·g^−1^) according to a previous report [24]. Platelet depletion was induced by intravenously injecting anti‐CD42b antibody (2 μg·g^−1^; R300; Emfret Analytics, Eibelstadt, Germany) or rat IgG isotype antibody (2 μg·g^−1^; Emfret Analytics) into WT mice 24 h before histone injection (75 μg·g^−1^) as previously described [25].

The survival time of mice treated with histones (75 μg·g^−1^) was recorded for 1 h. In addition, 1 h after histone injection (60 μg·g^−1^), we analyzed lung histology and measured platelet count, plasma MPO levels, and PLA percentage.

### Staining of NET‐like chromatin fibers

Neutrophils were isolated from the bone marrow of WT and Mac‐1^−/−^ mice using Percoll gradients (Sigma‐Aldrich). Isolated neutrophils were primed using tumor necrosis factor (TNF)‐α (Sigma‐Aldrich), seeded at 2.0 × 10^5^ cells/9.5 cm^2^, and incubated with RPMI medium, phorbol 12‐myristate 13‐acetate (PMA, 200 nm), calf thymus histones (900 μg·mL^−1^), or a mixture of calf thymus histones (900 μg·mL^−1^) and 5% PRP for 3 h at 37 °C. PRP was prepared from the whole blood of WT mice by centrifugation at 100 ***g*** for 10 min, and then, platelet‐poor plasma was prepared from this sedimentation via centrifugation at 1500 ***g*** for 10 min. Next, the neutrophils were washed with PBS and incubated with 5% BSA‐HBSS for 30 min at room temperature. After washing with PBS, neutrophils were incubated with Hoechst 33342 solution (Dojindo Molecular Technologies) and SYTOX Green (Thermo Fisher Scientific) for 30 min at room temperature in the dark. Images were captured at 200× magnification under a BZ‐X800 fluorescence microscope (Keyence). We measured the positive area of NET‐like chromatin fibers in the images using an Image Cytometer and bz‐x analysis software.

### Inhibition of NET formation

To inhibit NET formation, we injected DNase‐I (100 U/body; Promega, Madison, WI, USA) or saline into WT mice 5 min before histone injection (60 μg·g^−1^). The survival time of the mice was recorded for 1 h. Histological analysis, platelet count, and the percentage of PLAs were measured 1 h after histone injection.

### Statistical analysis

Data are shown as the mean ± standard deviation. Comparisons between multiple groups were performed using analysis of variance. Analyses between the two groups were performed using an unpaired *t*‐test (Student's *t*‐test). Time‐to‐event curves were plotted using the Kaplan–Meier method, and the groups were compared using the log‐rank test. In all statistical tests, a two‐sided *P*‐value < 0.05 was considered significant. spss v25.0 software (SPSS Inc, Chicago, IL, USA) was used for the statistical analyses.

## Results

### Depletion of neutrophils or platelets ameliorates histone‐induced ALI

**Fig. 1:**
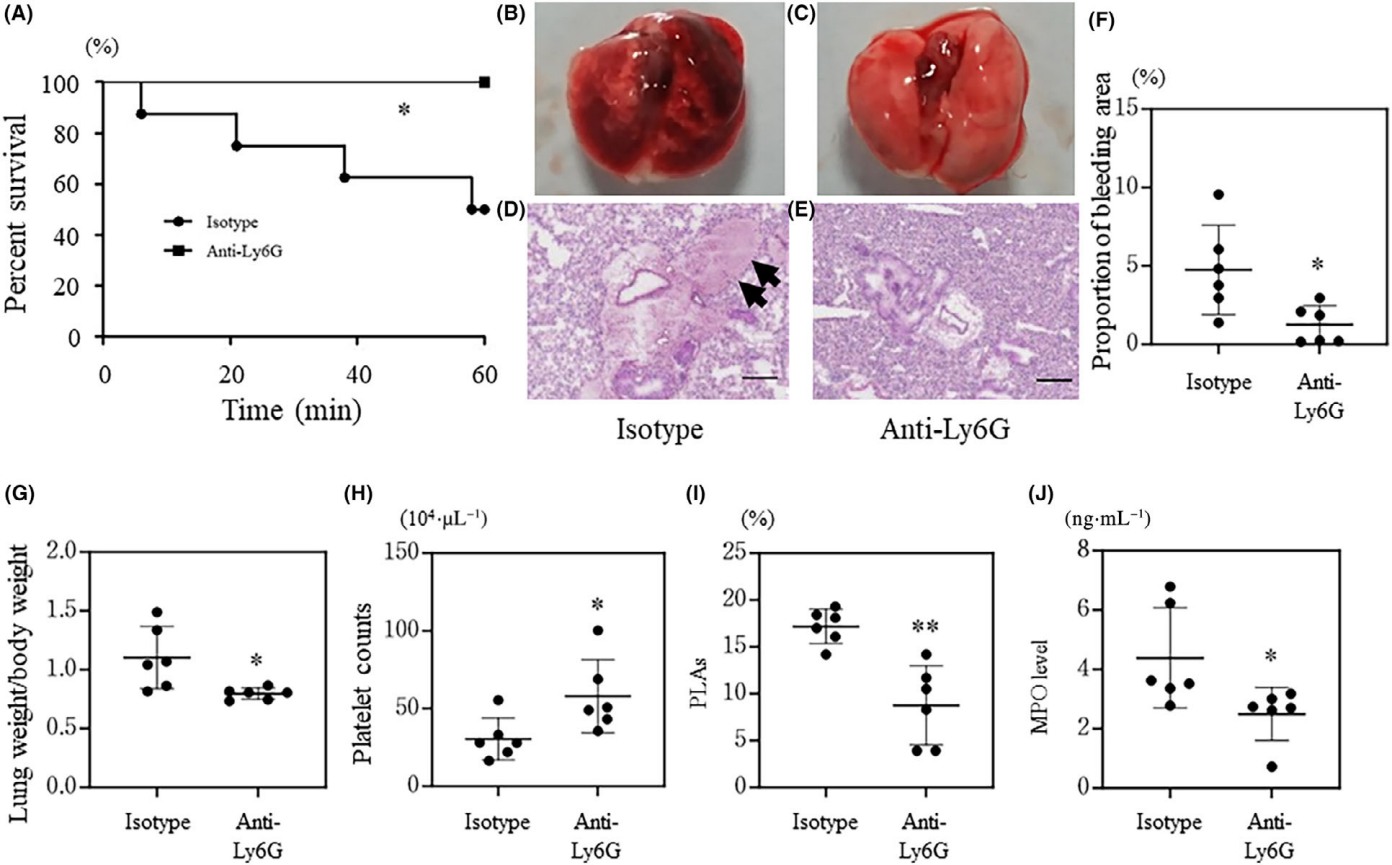
Depletion of neutrophils in wild‐type mice. C57BL/6J (wild‐type) mice pre‐treated with the anti‐Ly6G antibody or isotype IgG received a single tail vein injection of histones or saline. Panel (A) shows the survival time of each mouse, *n* = 8 per group. Panels (B–E) show macroscopic and microscopic (hematoxylin and eosin staining) findings. The black arrows indicate bleeding. The proportion of bleeding area in lung tissue is shown in panel (F), *n* = 6 per group. Lung weight/body weight (*n* = 6 per group), platelet counts (*n* = 6 per group), the proportion of platelet‐leukocyte aggregates (PLAs) (*n* = 6 per group), and myeloperoxidase (MPO) level (*n* = 6 per group) are shown in panels (G–J), respectively. The black bar indicates 200 μm. Values are shown as the mean SD (panels F–J). *P < 0.05, **P < 0.01 vs isotype (log‐link test, panel A; or Student's *t*‐test, panels F–J).

To clarify the role of leukocytes and platelets in ALI, we pre‐treated mice with anti‐Ly6G and anti‐CD42 antibodies to deplete the leukocytes and platelets before intravenous histone injection. Survival time after histone injection was significantly improved in neutrophil‐depleted mice pre‐treated with the anti‐Ly6G antibody compared to that in mice pre‐treated with isotype IgG (Fig. 1A). White blood cell counts after neutrophil depletion are shown in Fig. S1. Macroscopic and microscopic observations revealed that neutrophil depletion ameliorated pulmonary edema and lung bleeding in histone‐induced ALI (Fig. 1B–E). This result was further corroborated by a reduced bleeding area and lung weight in neutrophil‐depleted mice compared to those in control mice (Fig. 1F,G). Furthermore, the number of circulating platelets was higher and the proportion of PLAs was lower in neutrophil‐depleted mice than those in control mice (Fig. 1H,I). Plasma MPO levels, which are affected by neutrophil depletion, were lower in mice pre‐treated with the anti‐Ly6G antibody than those in mice pre‐treated with the isotype IgG (Fig. 1J).

**Fig. 2:**
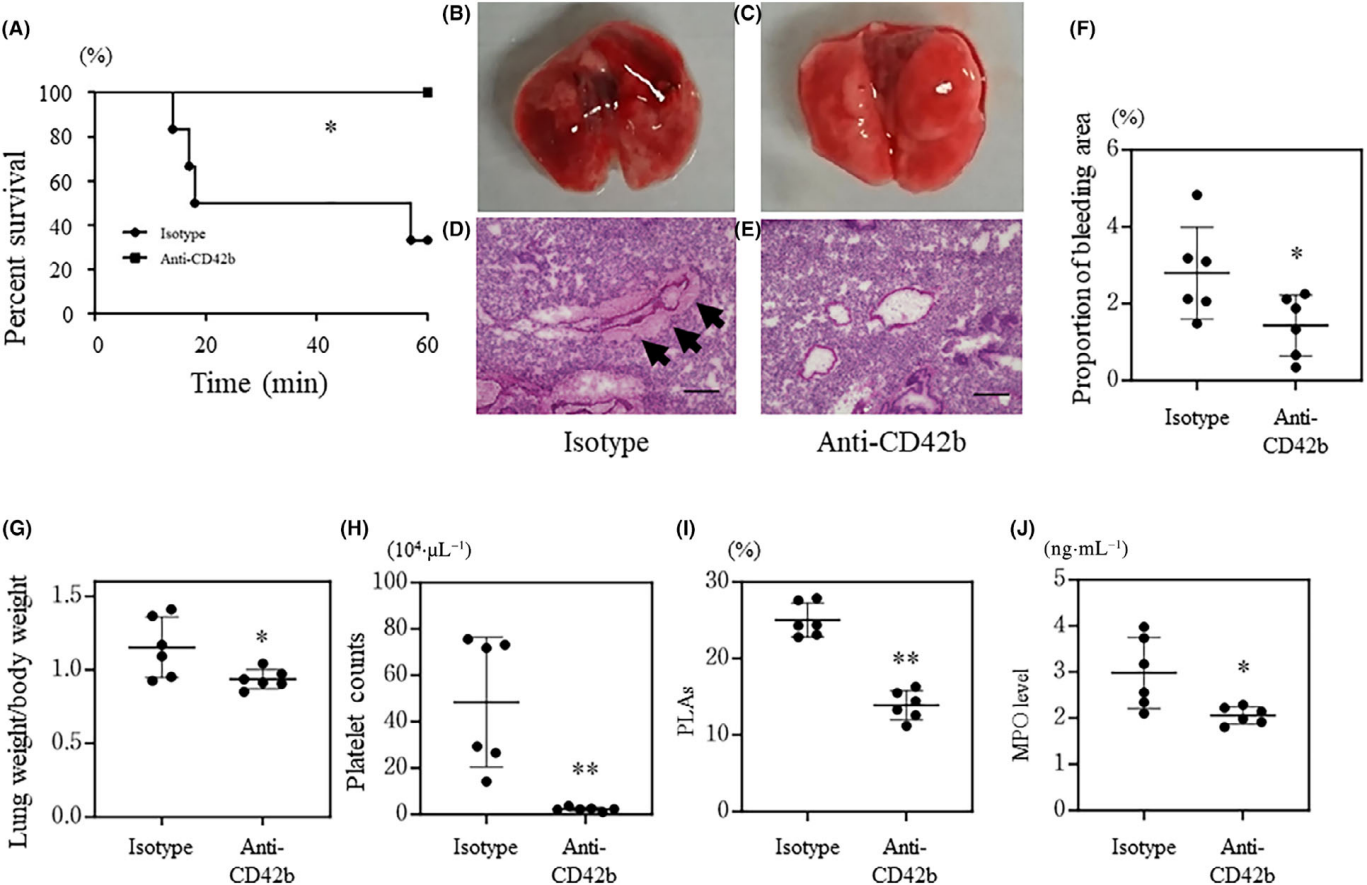
Depletion of platelets in wild‐type mice. Wild‐type mice pre‐treated with the anti‐CD42b antibody or isotype IgG received a single tail vein injection of histones (*n* = 6 per group). Panel (A) shows the survival time of each mouse. Panels (B–E) show macroscopic and microscopic (hematoxylin and eosin staining) findings. The black arrows indicate bleeding. The proportion of bleeding area in lung tissue is shown in panel (F). Lung weight/body weight, platelet counts, the proportion of platelet‐leukocyte aggregates (PLAs), and myeloperoxidase (MPO) level are shown in panels (G–J), respectively. The black bar indicates 200 μm. Values are shown as the mean SD (panels F–J). *P < 0.05, **P < 0.01 vs. isotype (log‐link test, panel A; or Student's *t*‐test, panels F–J).

Platelet depletion improved survival time (Fig. 2A) and macroscopic and microscopic findings in lung tissues (Fig. 2B–E) and decreased the bleeding area and lung weight (Fig. 2F,G). In addition, the number of circulating platelets and proportion of PLAs were lower in mice treated with the anti‐CD42b antibody than those in mice pre‐treated with the isotype IgG (Fig. 2H,I). Notably, MPO levels were decreased by platelet depletion (Fig. 2J).

### Mac‐1 deficiency ameliorates histone‐induced ALI and MPO elevation in plasma

**Fig. 3:**
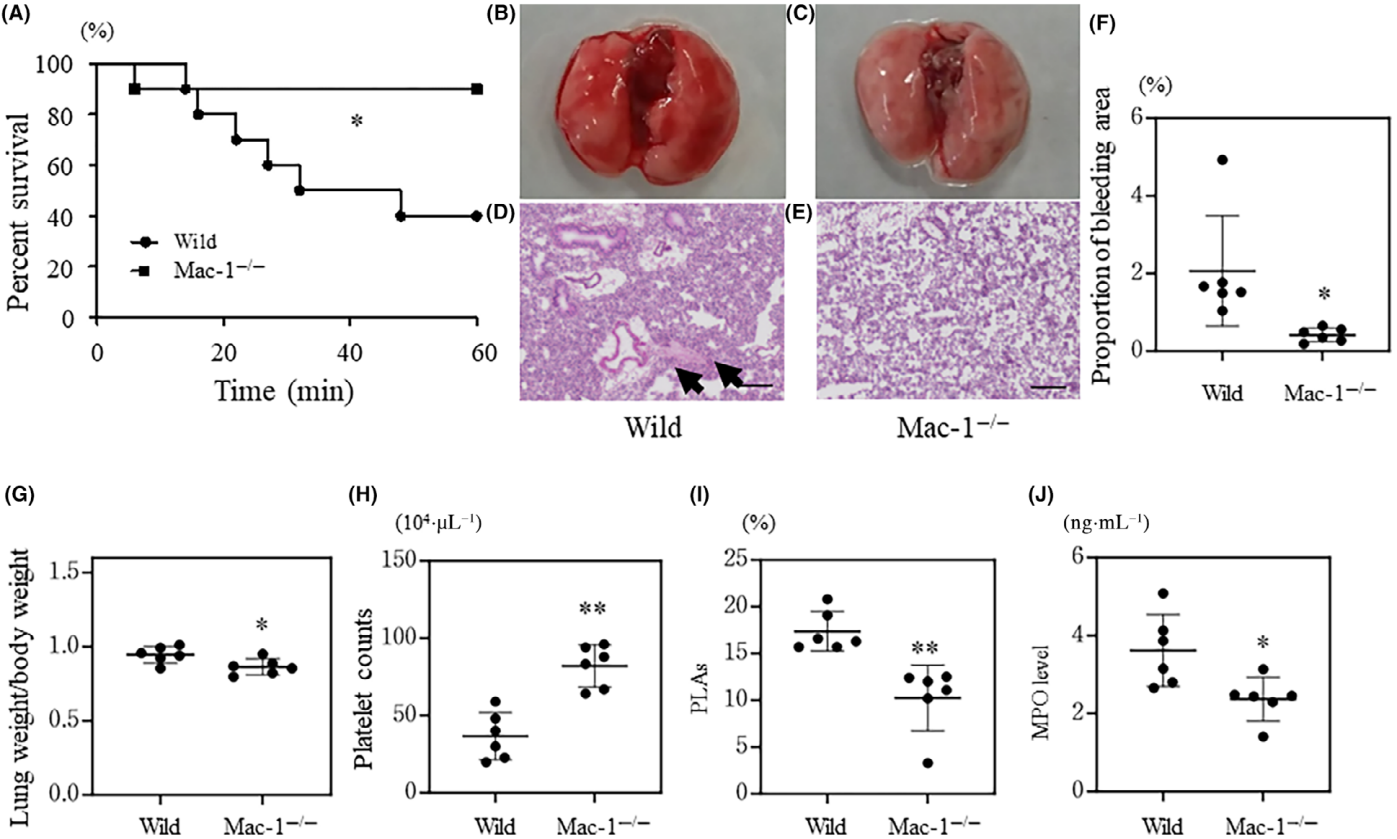
Comparison between wild‐type and Mac‐1^−/−^ mice treated with histones. Wild‐type and Mac‐1^−/−^ mice were treated with histones. Panel (A) shows the survival time of each mouse, *n* = 10 per group. Panels (B–E) show macroscopic and microscopic (hematoxylin and eosin staining) findings. The black arrows indicate bleeding. The proportion of bleeding area in lung tissue is shown in panel (F), *n* = 6 per group. Lung weight/body weight, platelet counts, the proportion of platelet‐leukocyte aggregates (PLAs), and myeloperoxidase (MPO) level are shown in panels (G–J) (*n* = 6 per group), respectively. The black bar indicates 200 μm. Values are shown as the mean SD (panels F–J). *P < 0.05, **P < 0.01 vs. wild‐type (log‐link test, panel A; or Student's *t*‐test, panels F–J).

Because Mac‐1‐expressing neutrophils have been implicated in the interaction between leukocytes and platelets, we assessed histone‐induced ALI in Mac‐1^−/−^ mice. The survival time of Mac‐1^−/−^ mice was greater than that of WT mice (Fig. 3A). Macroscopic and microscopic analyses demonstrated the amelioration of pulmonary edema and lung bleeding in Mac‐1^−/−^ mice compared to WT mice (Fig. 3B–G). In contrast to the higher number of circulating platelets in Mac‐1^−/−^ mice than in WT mice (Fig. 3H), the proportion of PLAs and MPO levels were lower in Mac‐1^−/−^ mice than in WT mice (Fig. 3I,J). These results indicate that Mac‐1 deficiency ameliorated histone‐induced ALI and decreased plasma MPO levels.

### Mac‐1 promotes the formation of NET‐like chromatin fibers in the presence of activated platelets

**Fig. 4:**
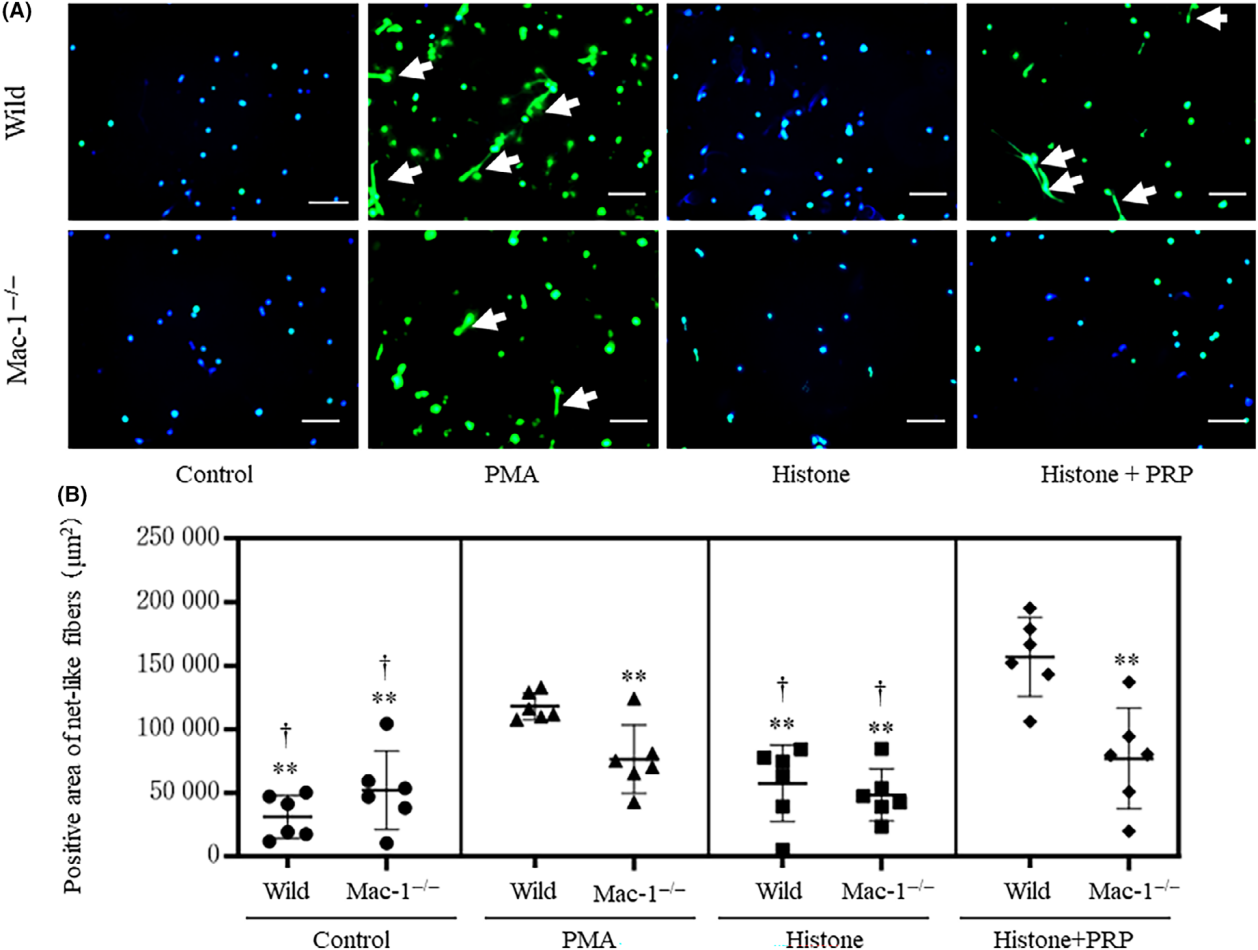
Net‐like chromatin fibers in neutrophils isolated from wild‐type and Mac‐1^−/−^ mice (*n* = 6 per group). Neutrophils isolated from WT or Mac‐1^−/−^ mice were incubated with RPMI medium, phorbol 12‐myristate 13‐acetate (PMA), histones, or a mixture of histones and 5% platelet‐rich plasma (PRP). Representative images of NET‐like fiber stains and the positive area of NET‐like chromatin fibers are shown in panel (A), and the analysis results of these areas are shown in panel (B). White arrows show NET‐like fibers, and the cell nucleus was stained by Hoechst 33342 solution (blue color). The white bar indicates 100 μm. Values are shown as the mean ± SD. **P < 0.01 vs. WT treated with histone + PRP, ^†^ *P* < 0.01 vs. WT treated with PMA (Tukey's test). Normality was validated by the Shapiro–Wilk test.

To investigate the role of Mac‐1 in the presence of activated neutrophils and platelets, we stained NET‐like chromatin fibers in BMNs using SYTOX Green. NETosis was not observed in neutrophils stimulated by histones, whereas NET‐like chromatin fibers were observed in the presence of platelet‐rich plasma (PRP) and histones (Fig. 4A). The area of NET‐like chromatin fibers was significantly decreased in BMNs from Mac‐1^−/−^ mice co‐stimulated with histones and PRP compared to that in BMNs from WT mice (Fig. 4B). Co‐stimulating with histones and platelet‐poor plasma did not increase the production of NET‐like chromatin fibers (Fig. S2). These results suggest that Mac‐1 promoted the formation of NET‐like chromatin fibers in the presence of activated platelets and histones.

### Pre‐treatment with DNase improves the survival of mice with histone‐induced ALI

**Fig. 5:**
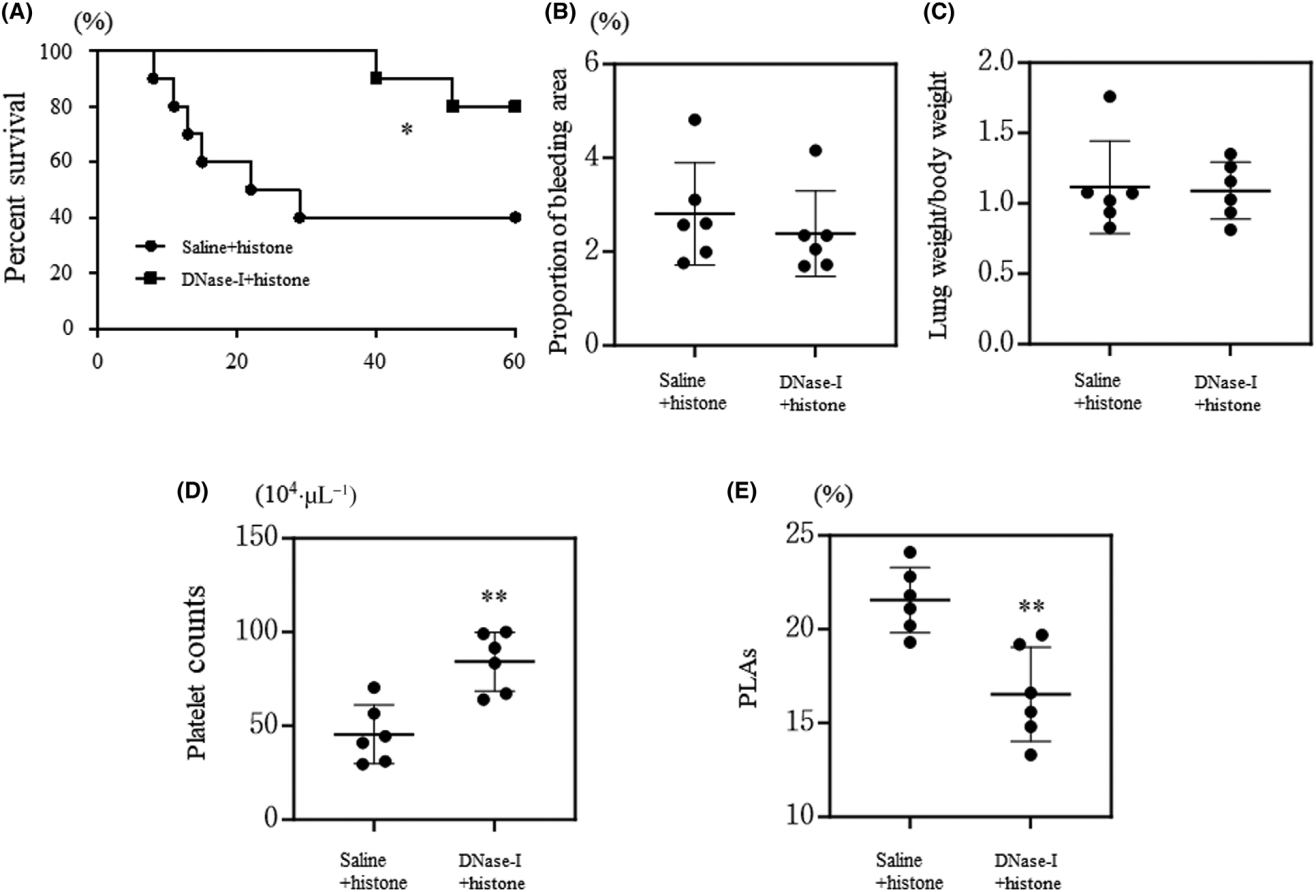
The effect of DNase in wild‐type mice treated with histones. Wild‐type mice pre‐treated with DNase‐I or saline received a single tail vein injection of histones. Survival time of each mouse (*n* = 10 per group), the proportion of bleeding area (*n* = 6 per group), lung weight/body weight (*n* = 6 per group), platelet counts (*n* = 6 per group), and proportion of platelet‐leukocyte aggregates (PLAs) (*n* = 6 per group) are shown in panels (A–E), respectively. Values are shown as the mean ± SD. *P < 0.05, **P < 0.01 vs. saline + histone (log‐link test, panel A; or Student's *t*‐test, panels B–E).

Although Mac‐1 promoted the formation of NET‐like chromatin fibers in activated BMNs, the role of NETs in histone‐induced ALI remains unclear. Therefore, we pre‐treated WT mice with DNase to clarify the role of NET formation in our ALI model. Pre‐treatment with DNase improved the survival time of mice administered with histones (Fig. 5A). However, there was no improvement in lung microscopic and macroscopic findings in mice pre‐treated with DNase (Fig. 5B,C). In contrast, pre‐treatment with DNase protected against the decrease in platelet count and PLA formation (Fig. 5D,E). Although DNase post‐treatment also inhibited PLA formation, it exacerbated lung injury and decreased the platelet count (Fig. S3). These results indicate that pre‐treatment with DNase improved the survival of mice with histone‐induced ALI.

## Discussion

Neutrophils play an essential role in the development of ALI by releasing NETs and various proinflammatory cytokines [4, 26]. Furthermore, excessive accumulation of leukocytes and platelets contributes to the development of ALI [27]. However, no studies have examined the interaction between leukocytes and platelets in a histone‐induced ALI model. The present study suggests that neutrophils and platelets activated by extracellular histones exacerbate ALI and that Mac‐1 plays an essential role in PLA and NET formation in histone‐induced ALI. Interactions between leukocytes and platelets contribute to the progression of systemic inflammation and immune reactions in ALI [28], while activated platelets promote immune cell recruitment and enhance leukocyte adhesion, phagocytosis, and intracellular killing through PLA formation [29]. PLAs secrete chemoattractants and granule proteins; therefore, PLA formation based on platelet and neutrophil recruitment increases vascular permeability and fluid leakage in the lung [28]. In the present study, to clarify the contribution of PLAs to histone‐induced ALI progression, we depleted neutrophils or platelets from WT mice. Neutrophil and platelet depletion drastically ameliorated histone‐induced ALI and decreased PLA formation. These results indicate that neutrophils or platelets might be therapeutic targets in the acute phase of lung injury, consistent with previous studies [30, 31].

In the present study, circulating MPO levels were decreased by neutrophil and platelet depletion, suggesting that PLA formation is associated with NETosis. Mac‐1 supports leukocyte adhesion to platelets through junctional adhesion molecule 3 [32], intercellular adhesion molecule‐2 [33], glycoprotein Ibа, and fibrinogen [34]. These interactions activate and promote neutrophil ligation and clustering, which enhances neutrophil activation via cell signaling mediated by Syk tyrosine kinases to form PLAs [35]. Various molecules are involved in PLA formation leading to NETosis, and high mobility group box 1 (HMGB1) is a key factor in the initiation of NETosis in the presence of activated neutrophils and platelets. Activated platelets upregulate the mitogen‐activated protein kinase/extracellular signal‐regulated kinase signaling pathway of neutrophils via HMGB1 and P‐selectin–P‐selectin glycoprotein ligand 1 interaction. This pathway promotes the release of MPO and neutrophil elastase (NE) from granules, and then NE drives histone processing [36]. However, whether Mac‐1 is associated with the above process triggered by activated platelets is unclear. In the present study, survival time after treatment of histone was higher in Mac‐1^−/−^ mice than in WT mice, and PLA formation and the release of MPO were lower in Mac‐1^−/−^ mice than in WT mice. Furthermore, the formation of NET‐like chromatin fibers was lower in BMNs from Mac‐1^−/−^ mice than that in BMNs from WT mice in the presence of PRP, but the differences were not observed in the presence of PMA. These results suggest that Mac‐1 is associated with the exacerbation of histone‐induced ALI and activates platelet‐induced NET formation. In previous studies, Mac‐1 exhibited a protective role against CLP‐induced lung edema [17], suggesting that the recruitment of neutrophils via Mac‐1 to the lung contributed to the alleviation of PAMP‐induced ALI. In contrast, neutrophil depletion attenuated lung injury in a histone‐induced ALI model in the present study. Recently, Siegel *et al*. [19], demonstrated similar effects of neutrophils in PAMP‐induced ALI, and our results agree with this report. Although our results partially contradict the aforementioned study, they suggest that the role of neutrophil recruitment differs between PAMP‐ and DAMP‐induced ALI.

In the present study, to elucidate the pathological role of NET formation in histone‐induced ALI, we inhibited NET formation in WT mice by pre‐treatment with DNase. The survival time and consumption of platelets were improved by pre‐treatment with DNase and PLA formation was decreased. However, macroscopic and microscopic findings of the lungs were not improved by pre‐treatment with DNase. PLAs are associated with the development of ALI resulting from the release of elastase and MPO [37]. Our results suggest that the inhibition of NET formation decreases PLA formation but does not ameliorate lung hemorrhaging. In addition, depending on the timing of the inhibition, it may exacerbate lung damage. DNase‐I alleviates diffuse alveolar hemorrhaging in coagulation disorders caused by systemic lupus erythematosus [38]. Moreover, a histone‐induced lethal thrombosis animal model showed severe hyperfibrinolysis in the acute phase [23, 39]. Therefore, a dual strategy is required to control the coagulation and fibrinolysis systems to ameliorate histone‐induced ALI. NETs control local hemorrhaging by activating and recruiting platelets. As a definite amount of NETosis is needed for the proper hemostasis, NETs inhibition might exacerbate lung damage depending on the timing. However, the depletion of neutrophils or platelets drastically improved the mortality and pathological findings of histone‐induced ALI in our study, suggesting that inhibiting the activation of neutrophils or platelets might be an ideal strategy to alleviate ALI induced by DAMPs. As Mac‐1 controls the activation of neutrophils or platelets, inhibition of Mac‐1 function in ALI may be a therapeutic strategy for preventing NETs formation in patients with ALI.

In conclusion, the present study revealed that Mac‐1 is associated with the exacerbation of histone‐induced ALI and the promotion of NET formation in the presence of activated platelets.

## Conflict of interest

The authors declare no conflict of interest.

### Peer review

The peer review history for this article is available at https://www.webofscience.com/api/gateway/wos/peer‐review/10.1002/2211‐5463.13779.

## Author contributions

TM contributed to the study conception and design and drafted the manuscript. FN performed the experiments, analyzed, and interpreted the data. SY, KT, KF, and SM contributed to the study design and reviewed the manuscript. NT contributed to the study conception and design, supervised the execution of the study, and reviewed the manuscript.

## Supporting information


**Fig. S1.** Neutrophil counts after neutrophil depletion.
**Fig. S2.** Net‐like chromatin fibers in neutrophils isolated from wild‐type treated with platelet‐poor plasma and platelet‐rich plasma.
**Fig. S3.** The effect of post‐treatment with DNase in wild‐type mice treated with histones.

## Data Availability

The datasets generated and/or analyzed during the current study are available from the corresponding author upon reasonable request.

## References

[1] Bellani G , Laffey JG , Pham T , Fan E , Brochard L , Esteban A , Gattinoni L , van Haren F , Larsson A , McAuley DF *et al*. (2016) Epidemiology, patterns of care, and mortality for patients with acute respiratory distress syndrome in intensive care units in 50 countries. JAMA 315, 788–800. 26903337 10.1001/jama.2016.0291

[2] Mitchell WB (2020) Thromboinflammation in COVID‐19 acute lung injury. Paediatr Respir Rev 35, 20–24. 32653469 10.1016/j.prrv.2020.06.004 PMC7289106

[3] Ranieri VM , Rubenfeld GD , Thompson BT , Ferguson ND , Caldwell E , Fan E , Camporota L and Slutsky AS (2012) Acute respiratory distress syndrome: the Berlin definition. JAMA 307, 2526–2533. 22797452 10.1001/jama.2012.5669

[4] Butt Y , Kurdowska A and Allen TC (2016) Acute lung injury: a clinical and molecular review. Arch Pathol Lab Med 140, 345–350. 27028393 10.5858/arpa.2015-0519-RA

[5] Ito T (2014) PAMPs and DAMPs as triggers for DIC. J Intensive Care 2, 67. 10.1186/s40560-014-0065-0 PMC4336279 25705424

[6] Herter JM , Rossaint J and Zarbock A (2014) Platelets in inflammation and immunity. J Thromb Haemost 12, 1764–1775. 25224706 10.1111/jth.12730

[7] Bosmann M , Grailer JJ , Ruemmler R , Russkamp NF , Zetoune FS , Sarma JV , Standiford TJ and Ward PA (2013) Extracellular histones are essential effectors of C5aR‐ and C5L2‐mediated tissue damage and inflammation in acute lung injury. FASEB J 27, 5010–5021. 23982144 10.1096/fj.13-236380 PMC3834784

[8] Middleton EA , He XY , Denorme F , Campbell RA , Ng D , Salvatore SP , Mostyka M , Baxter‐Stoltzfus A , Borczuk AC , Loda M *et al*. (2020) Neutrophil extracellular traps contribute to immunothrombosis in COVID‐19 acute respiratory distress syndrome. Blood 136, 1169–1179. 32597954 10.1182/blood.2020007008 PMC7472714

[9] Xu Z , Huang Y , Mao P , Zhang J and Li Y (2015) Sepsis and ARDS: the dark side of histones. Mediators Inflamm 2015, 205054. 26609197 10.1155/2015/205054 PMC4644547

[10] Fuchs TA , Brill A , Duerschmied D , Schatzberg D , Monestier M , Myers DD Jr , Wrobleski SK , Wakefield TW , Hartwig JH and Wagner DD (2010) Extracellular DNA traps promote thrombosis. Proc Natl Acad Sci USA 107, 15880–15885. 20798043 10.1073/pnas.1005743107 PMC2936604

[11] Saffarzadeh M , Juenemann C , Queisser MA , Lochnit G , Barreto G , Galuska SP , Lohmeyer J and Preissner KT (2012) Neutrophil extracellular traps directly induce epithelial and endothelial cell death: a predominant role of histones. PLoS One 7, e32366. 22389696 10.1371/journal.pone.0032366 PMC3289648

[12] Martinod K and Deppermann C (2021) Immunothrombosis and thromboinflammation in host defense and disease. Platelets 32, 314–324. 32896192 10.1080/09537104.2020.1817360

[13] Rondina MT , Weyrich AS and Zimmerman GA (2013) Platelets as cellular effectors of inflammation in vascular diseases. Circ Res 112, 1506–1519. 23704217 10.1161/CIRCRESAHA.113.300512 PMC3738064

[14] Wang Y , Gao H , Shi C , Erhardt PW , Pavlovsky A , Soloviev DA , Bledzka K , Ustinov V , Zhu L , Qin J *et al*. (2017) Leukocyte integrin Mac‐1 regulates thrombosis via interaction with platelet GPIbα. Nat Commun 8, 15559. 28555620 10.1038/ncomms15559 PMC5477519

[15] Dunne JL , Ballantyne CM , Beaudet AL and Ley K (2002) Control of leukocyte rolling velocity in TNF‐alpha‐induced inflammation by LFA‐1 and Mac‐1. Blood 99, 336–341. 11756189 10.1182/blood.v99.1.336

[16] Silva JC , Rodrigues NC , Thompson‐Souza GA , Muniz VS , Neves JS and Figueiredo RT (2020) Mac‐1 triggers neutrophil DNA extracellular trap formation to *Aspergillus fumigatus* independently of PAD4 histone citrullination. J Leukoc Biol 107, 69–83. 31478251 10.1002/JLB.4A0119-009RR

[17] Liu JR , Han X , Soriano SG and Yuki K (2014) The role of macrophage 1 antigen in polymicrobial sepsis. Shock 42, 532–539. 25075642 10.1097/SHK.0000000000000250

[18] Asaduzzaman M , Zhang S , Lavasani S , Wang Y and Thorlacius H (2008) LFA‐1 and MAC‐1 mediate pulmonary recruitment of neutrophils and tissue damage in abdominal sepsis. Shock 30, 254–259. 18197144 10.1097/shk.0b013e318162c567

[19] Siegel PM , Przewosnik AS , Wrobel J , Heidt T , Moser M , Peter K , Bode C , Diehl P and Bojti I (2022) An activation specific anti‐Mac‐1 designed ankyrin repeat protein improves survival in a mouse model of acute lung injury. Sci Rep 12, 6296. 35428807 10.1038/s41598-022-10090-6 PMC9012056

[20] Zhao F , Ma Q , Yue Q and Chen H (2022) SARS‐CoV‐2 infection and lung regeneration. Clin Microbiol Rev 35, e0018821. 35107300 10.1128/cmr.00188-21 PMC8809385

[21] Skendros P , Mitsios A , Chrysanthopoulou A , Mastellos DC , Metallidis S , Rafailidis P , Ntinopoulou M , Sertaridou E , Tsironidou V , Tsigalou C *et al*. (2020) Complement and tissue factor‐enriched neutrophil extracellular traps are key drivers in COVID‐19 immunothrombosis. J Clin Invest 130, 6151–6157. 32759504 10.1172/JCI141374 PMC7598040

[22] Nagano F , Mizuno T , Imai M , Takahashi K , Tsuboi N , Maruyama S and Mizuno M (2022) Expression of a Crry/p65 is reduced in acute lung injury induced by extracellular histones. FEBS Open Bio 12, 192–202. 10.1002/2211-5463.13322 PMC8727949 34709768

[23] Mizuno T , Yoshioka K , Mizuno M , Shimizu M , Nagano F , Okuda T , Tsuboi N , Maruyama S , Nagamatsu T and Imai M (2017) Complement component 5 promotes lethal thrombosis. Sci Rep 7, 42714. 28205538 10.1038/srep42714 PMC5311936

[24] Shi Y , Tsuboi N , Furuhashi K , Du Q , Horinouchi A , Maeda K , Kosugi T , Matsuo S and Maruyama S (2014) Pristane‐induced granulocyte recruitment promotes phenotypic conversion of macrophages and protects against diffuse pulmonary hemorrhage in Mac‐1 deficiency. J Immunol 193, 5129–5139. 25281714 10.4049/jimmunol.1401051

[25] Hirahashi J , Hishikawa K , Kaname S , Tsuboi N , Wang Y , Simon DI , Stavrakis G , Shimosawa T , Xiao L , Nagahama Y *et al*. (2009) Mac‐1 (CD11b/CD18) links inflammation and thrombosis after glomerular injury. Circulation 120, 1255–1265. 19752320 10.1161/CIRCULATIONAHA.109.873695 PMC2780001

[26] Williams AE and Chambers RC (2014) The mercurial nature of neutrophils: still an enigma in ARDS? Am J Physiol Lung Cell Mol Physiol 306, L217–L230. 24318116 10.1152/ajplung.00311.2013 PMC3920201

[27] Shannon O (2021) The role of platelets in sepsis. Res Pract Thromb Haemost 5, 27–37. 33537527 10.1002/rth2.12465 PMC7845078

[28] de Stoppelaar SF , van 't Veer C and van der Poll T (2014) The role of platelets in sepsis. Thromb Haemost 112, 666–677. 24966015 10.1160/TH14-02-0126

[29] Peters MJ , Dixon G , Kotowicz KT , Hatch DJ , Heyderman RS and Klein NJ (1999) Circulating platelet‐neutrophil complexes represent a subpopulation of activated neutrophils primed for adhesion, phagocytosis and intracellular killing. Br J Haematol 106, 391–399. 10460597 10.1046/j.1365-2141.1999.01553.x

[30] Russwurm S , Vickers J , Meier‐Hellmann A , Spangenberg P , Bredle D , Reinhart K and Lösche W (2002) Platelet and leukocyte activation correlate with the severity of septic organ dysfunction. Shock 17, 263–268. 11954824 10.1097/00024382-200204000-00004

[31] Gawaz M , Dickfeld T , Bogner C , Fateh‐Moghadam S and Neumann FJ (1997) Platelet function in septic multiple organ dysfunction syndrome. Intensive Care Med 23, 379–385. 9142575 10.1007/s001340050344

[32] Santoso S , Sachs UJ , Kroll H , Linder M , Ruf A , Preissner KT and Chavakis T (2002) The junctional adhesion molecule 3 (JAM‐3) on human platelets is a counterreceptor for the leukocyte integrin Mac‐1. J Exp Med 196, 679–691. 12208882 10.1084/jem.20020267 PMC2194005

[33] Kuijper PH , Gallardo Tores HI , Lammers JW , Sixma JJ , Koenderman L and Zwaginga JJ (1998) Platelet associated fibrinogen and ICAM‐2 induce firm adhesion of neutrophils under flow conditions. Thromb Haemost 80, 443–448. 9759625

[34] Li J , Kim K , Jeong SY , Chiu J , Xiong B , Petukhov PA , Dai X , Li X , Andrews RK , Du X *et al*. (2019) Platelet protein disulfide isomerase promotes glycoprotein Ibα‐mediated platelet‐neutrophil interactions under thromboinflammatory conditions. Circulation 139, 1300–1319. 30586735 10.1161/CIRCULATIONAHA.118.036323 PMC6464389

[35] Futosi K , Fodor S and Mócsai A (2013) Neutrophil cell surface receptors and their intracellular signal transduction pathways. Int Immunopharmacol 17, 638–650. 23994464 10.1016/j.intimp.2013.06.034 PMC3827506

[36] Papayannopoulos V (2018) Neutrophil extracellular traps in immunity and disease. Nat Rev Immunol 18, 134–147. 28990587 10.1038/nri.2017.105

[37] Grommes J , Alard JE , Drechsler M , Wantha S , Mörgelin M , Kuebler WM , Jacobs M , von Hundelshausen P , Markart P , Wygrecka M *et al*. (2012) Disruption of platelet‐derived chemokine heteromers prevents neutrophil extravasation in acute lung injury. Am J Respir Crit Care Med 185, 628–636. 22246174 10.1164/rccm.201108-1533OC PMC3326286

[38] Jarrot PA , Tellier E , Plantureux L , Crescence L , Robert S , Chareyre C , Daniel L , Secq V , Garcia S , Dignat‐George F *et al*. (2019) Neutrophil extracellular traps are associated with the pathogenesis of diffuse alveolar hemorrhage in murine lupus. J Autoimmun 100, 120–130. 30930069 10.1016/j.jaut.2019.03.009

[39] Nakahara M , Ito T , Kawahara K , Yamamoto M , Nagasato T , Shrestha B , Yamada S , Miyauchi T , Higuchi K , Takenaka T *et al*. (2013) Recombinant thrombomodulin protects mice against histone‐induced lethal thromboembolism. PLoS One 8, e75961. 24098750 10.1371/journal.pone.0075961 PMC3786915

