# Supplementary material for: Macrophage‐1 antigen exacerbates histone‐induced acute lung injury and promotes neutrophil extracellular trap formation
Source: FEBS Open Bio. 2024 Feb 15;14(4):574–83. doi: 10.1002/2211-5463.13779 (PMC10988669; doi:10.1002/2211-5463.13779)
Supplement: Supplementary file 1 — Fig. S1. Neutrophil counts after neutrophil depletion. Fig. S2. Net‐like chromatin fibers in neutrophils isolated from wild‐type treated with platelet‐poor plasma and platelet‐rich plasma. Fig. S3. The effect of post‐treatment with DNase in wild‐type mice treated with histones. [file FEB4-14-574-s001.docx]

**Macrophage-1 antigen exacerbates histone-induced acute lung injury and promotes neutrophil extracellular trap formation**

Tomohiro Mizuno^1*^, Fumihiko Nagano^2^, Kazuo Takahashi^3^, Shigeki Yamada^1^, Kazuhiro Fruhashi^2^, Shoichi Maruyama^2^, and Naotake Tsuboi^4^

^1^Department of Pharmacotherapeutics and informatics, Fujita Health University School of Medicine, Toyoake, Japan

^2^Department of Nephrology, Nagoya University School of Medicine, Nagoya, Japan

^3^Department of Biomedical Molecular Sciences, Fujita Health University School of Medicine, Toyoake, Japan

^4^Department of Nephrology, Fujita Health University School of Medicine, Toyoake, Japan

**^*^**These authors equally contributed to this work.

**Corresponding author**

Tomohiro Mizuno, Ph.D.

Department of Pharmacotherapeutics and informatics, Fujita Health University

1-98 Dengakugakubo, Kutsukake-cho, Toyoake, 470-1192, Japan

Tel: (+81)562-93-2493;

Fax: (+81)562-93-4593

E-mail: [tomohiro.mizuno@fujita-hu.ac.jp](mailto:tomohiro.mizuno@fujita-hu.ac.jp)


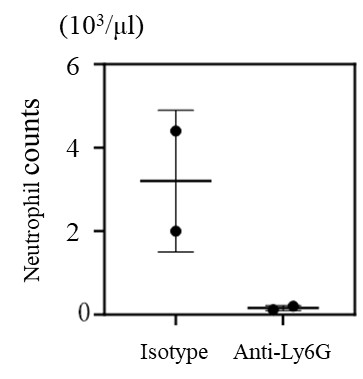


**Supplemental figure 1.** Neutrophil counts after neutrophil depletion

We intravenously injected anti-Ly6G antibody (Clone 1A8; Bioxcell, NH, USA; 250 µg/body) or rat IgG2 isotype antibody (250 µg/body; Bioxcell) into WT mice 48 h before neutrophil count (n=2). PE-conjugated rat anti-mouse CD11b antibody (clone

M1/70; BioLegend), rat anti-mouse CD16/32 antibody (clone 93; Proteintech, Rosemont, IL, USA), Zombie Aqua™ Fixable Viability Kit (BioLegend), and precision count beads (BioLegend) were used to count neutrophil in whole blood samples. Values are shown as the mean ± SD.


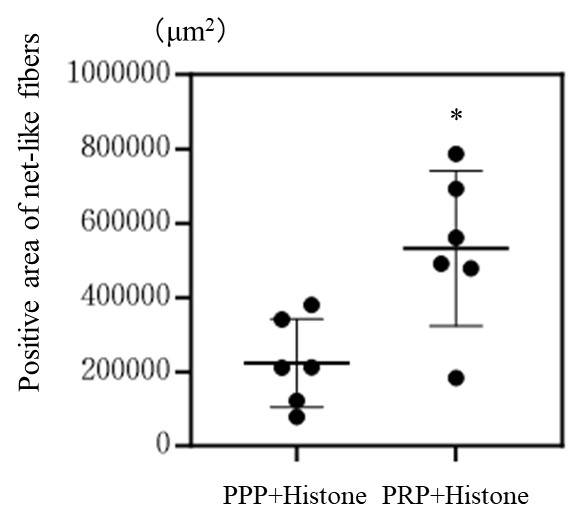


**Supplemental figure 2.** Net-like chromatin fibers in neutrophils isolated from wild-type treated with platelet-poor plasma and platelet-rich plasma.

Neutrophils isolated from WT or Mac-1^-/-^mice were incubated with a mixture of histones and 5% platelet-poor plasma (PPP) or 5% platelet-rich plasma (PRP). Values are shown as the mean ± SD. *P < 0.05 vs. PPP+histone (Student’s *t*-test).


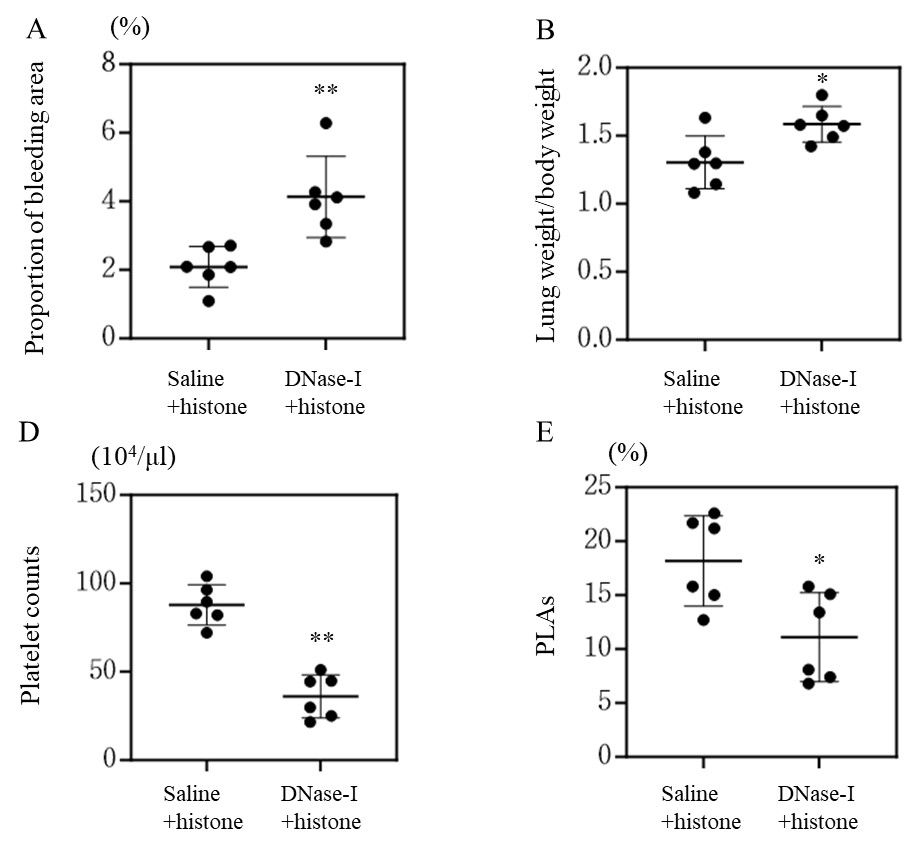


**Supplemental figure 3.** The effect of post-treatment with DNase in wild-type mice treated with histones.

Wild-type mice post-treated with DNase-I or saline received a single tail vein injection of histones. The proportion of bleeding area (n=6 per group), lung weight/body weight (n=6 per group), platelet counts (n=6 per group), and proportion of platelet-leukocyte aggregates (PLAs) (n=6 per group) are shown in panels A to D, respectively. Values are shown as the mean ± SD. *P < 0.05, ** P <0.01 vs. saline+histone (Student’s t-test, panel A to D).
